# Supplementary material for: Surveillance and control efficacy of the Bergerac, France, 2025 chikungunya outbreak
Source: PLoS Negl Trop Dis. 2026 Apr 20;20(4):e0014184. doi: 10.1371/journal.pntd.0014184 (PMC13124055; doi:10.1371/journal.pntd.0014184)
Supplement: Attachment — Submitted filename: Response to Reviewers.docx [file pntd.0014184.s002.docx]

**Response to Reviewers (PNTD-D-25-02091)**

Thanks to the editor and reviewers for their time and expertise in reviewing our manuscript. Below, we have pasted all comments in black and responded in full in red.

**Reviewers' Comments:**

**Methods**

Reviewer #1: I feel they have tried their best to analyse their data after the outbreak has occurred. It is not a research project. Perhaps they are trying to do a model.

We clearly state that we use a model throughout the paper.

Reviewer #2: The authors used a mathematical model to evaluate the effectiveness of mosquito control activities during a chikungunya outbreak in Bergerac, France. I am not an expert in this modeling, and the applied method needs to be evaluated by an expert in this field. However, the study design the authors used is adequate to address the objectives.

Thank you.

Reviewer #3: The material and method require improvement to be clearer.

We have addressed these issues. Thank you for the specific comments listed below.

**Results**
Reviewer #1: This is okay. Because they are modelling after something has occurred. If they had read widely they would have noticed that there are proactive methods that have been suggested for vector surveillance especially for dengue

With respect, we do not think that the reviewer has understood the context here. Yes, it is widely known that proactive methods of vector control can reduce the health burden on mosquito-borne diseases, particularly in endemic regions of the world. However, in much of Europe, where *Aedes*-borne disease risk is lower than in Brazil, say, vector control is only conducted once an outbreak is detected. This is decided by national health authorities, and it is not the role of independent research scientists to change policy (though they can provide evidence and tools to help policy makers). In this study, we can only report and analyse what occurred to provide evidence on the effectiveness of the strategies employed in this context.

Reviewer #2: The results presented correspond to the analysis plan and are clearly and completely presented. The figures are ok!

Thanks.

Reviewer #3: The obtained results are very relevant, however the material require revision in order to interpret the results according to the real situation (biology of the species) and not only according to the model analyses.

We have addressed these issues. Thank you for the specific comments listed below.

**Conclusions**
Reviewer #1: I feel more in depth analysis could have been carried out.

We are unable to do this as the reviewer has not specified what the additional analyses should be. As stated by both Reviewers 2 & 3, the analyses are relevant and appropriate.

Reviewer #2: The conclusions of the manuscript are supported by the methods applied and the results obtained. Its relevance lies in the need to conduct studies evaluating the effectiveness of mosquito control measures during arbovirus outbreaks. It's also relevant because few studies like this have been conducted.

Thanks, especially for noting the novelty of the work.

Reviewer #3: The conclusions follow what was presented in the results.

Thanks.

**Editorial and Data Presentation Modifications?**
Reviewer #1: This is not a problem

Reviewer #2: 1 - Background

- line 44: I would not say "small outbreaks". Outbreaks are outbreaks!
Word deleted

2 - Methods

- lines 103 to 105: to inform the case and probable case definitions for chikungunya in the French surveillance system.

Definitions and citation now given.

- line 168: the velocity and direction of the wind could be considered as covariates in the used model?

The model is a deterministic mathematical model and not statistical. It is not possible to include these variables as there is insufficient data to parameterise such vector dispersal and its dependence on wind conditions.

3 - Results

- line 221: I wouldn't say a "substantial effect", instead I would say a "moderate effect".

Changed as suggested.
- lines 257 to 267: I think this paragraph would be better in the Discussion item.

We prefer the paragraph in the Results since it is essentially a summary of the results, rather than a wider discussion.

4 - Discussion

- line 271: I wouldn't say "substantially reduce autochthonous case numbers". I would use 'moderately' instead of 'substantially'.

Changes as suggested.

Reviewer #3: (No Response)

NA

**Summary and General Comments**

Reviewer #1: Here I feel that the authors have not read widely. It has already been shown that fogging after cases are reported are not effective. There is no correlation between cases and mosquitoes. It has been established that cases occur lag of one to 2 weeks after infected mosquitoes are found. RCT have been conducted but this has not been quoted. The authors talk about detecting the virus in wastewater. It would be more practical to detect virus in mosquitoes as simple traps can collect the adult mosquitoes. Such studies have been published since 2011.

As both Reviewers 2 & 3 agree, the work is novel, relevant and highly valuable. It is our belief that this reviewer has misunderstood the work and context presented here. Furthermore, their comments are not sufficiently specific for us to respond to in detail.

Reviewer #2: (No Response)

NA.

Reviewer #3:

Dear Authors,
Regarding the increasing risk of vector-borne diseases to the public health globally, the studies which could offer answers to the numerous questions that about it, are considered highly valuable. The large experience that countries as Italy and France gained through to fight with the autochthonous cases in the last outbreak that they faced with, represent a good source of data and lessons learnt. The manuscript titled “Surveillance and control efficacy of the Bergerac, France, 2025 chikungunya outbreak”is an interesting and very relevant material that is worth of publishing as a solid evidence of mosquito control quality assessment during the outbreak.

Thank you for your assessment of our work, particularly noting the novelty and analysis of the evidence that it gives.

However, there are issues that need to be addressed before the submitted material is published. First of all, the title could be significantly improved. For example: it is not control efficacy of the Bergerac but it is quality control of mosquito treatment in Bergerac or evaluation of mosquito control etc… Please be specific and precise.

The study examines how well control methods reduce disease transmission, which is the definition of efficacy. Furthermore, since public awareness was raised alongside the vector control, and we are unable to delineate between the two, a the more generic “control methods” phrase is best suited for the title. Therefore, the title is indeed specific and precise.

Very important issues are: In the material and method section, is very difficult to follow what was done and why the procedure was changed. That should be explained in details. Why did the treatment start delayed? And why so delayed? If the outbreak was detected in June and treatment was carried out two months later, it means that you could have many, many infected mosquitoes flying around. The Ae. albopictus can survive for a long period of time (few months), so if adults avoided adulticiding they could survive the whole given period that you mentioned. And since the larvae were not in a focus, new adults were emerging again and again.

As stated in the manuscript, the first case (start of symptom onset) was confirmed on 6^th^ August 2025. However, subsequent testing identified earlier cases that were unknown at the time of the first detection, which was described elsewhere. We have made further clarifications in the Methods section to address this to help the reader understand the context.

The authors were saying that larviciding did not give good effect but the operators did not perform it in the satisfying level as the authors stated. If the larviciding was not done intensively, there were always new adults coming from the water and representing new threat by spreading virus as said above.

We have now repeated elsewhere in the manuscript that the use of larvicides was limited. We have been careful not to suggest that larvicide control, in general, was ineffective. As the reviewer points out, the larvicide treatments were not intensive, if they were, then we may have indeed seen a different outcome.

If the procedure was not identical at the beginning of the treatments and later, we cannot compare these two periods when the treatment was done. Additionally, there is cumulative effect of treatment. If the operators did not intensively treated between June and August, and then treated intensively in August but without treating juveniles, it is clear that why will need longer period to suppress the population.

It is not true that we cannot compare early (limited) and late (intensive) control periods. If the early control had a significant impact on the outbreak, then we argue that the model predictions would have substantially deviated from the reported case numbers. This did not occur, and therefore we conclude that the early control period had little effect in reducing cases. Furthermore, the control was done reactively, in line with French policy, and therefore there was no control before the first case was identified (6^th^ August). The Reviewer’s assessment for the latter need for long periods of (intense) control is exactly the point of the paper. However, due to the lagged effects of control on the mosquito populations, alongside impacts of local climate variability, the observed case numbers alone cannot support this conclusion, and hence the need for the modelling analysis that we provide here.

In the M&M should also be explain what data bases were used when the authors took into consideration the biology of the vector. Did you use your own data base? What about biting rates? How was that measured? Or is that estimated? Please give as many as possible details to provide good understanding and reproducibility of this study.

The model framework is described in full in <https://www.nature.com/articles/s41467-024-52144-5>, which describes transmission of DENV by *Ae. albopictus*. This publication contains all references to published studies used to parameterise the vector biology. Since this framework has already been published and detailed in full elsewhere, we feel it is not necessary to repeat the methodology akin to citing a statistical technique. Additional parameters are required for transmission of CHIKV, for which we give references to. We have edited the Methods section to improve the clarity. In this manuscript it is our intention to give the reader a solid, high-level understanding of the model without being distracted by hundreds of already published equations. We give full details on the methods used to apply the model to the Bergerac CHIKV outbreak and provide the model code to reproduce our outputs.

Please also find my specific comments:

L45 Please add year and author who described the species, when the species is mentioned for the first time in text.

Done.

L53 When you say highly competent, it is necessary to specify pathogen. Because it is not competent for all pathogens.

Good point. We have deleted the “highly competent” part to improve readability and accuracy.

L57 It is not the level, but the number. Same comment for L64.

L57 changed, as suggested. We believe that the second use of level is correct.

L60 Instead of “high levels of travel” it would be more suitable to say “high frequency of travelling”

Changed.

L79 The authors said “many” but gave only one citation as a example. There should be give at least 5 citations if you say “many”

Changed, as suggested.

L96 Please replace “where” with “if” .

Changed, as suggested.

L115 Please explain the reasons for the gap. It is crucial for readers to understand the whole concept.

Explained, as suggested.

L119-124 The sentence is too long and difficult to follow. Please simplify and be more specific about what was done. From the written text it sounds like interventions represented combination of adulticiding, suitability of environment, larviciding , presence of vectors… but it is not clear what exactly was carried out.

Sentence edited, as suggested.

L124 was done instead of were done

Changed, as suggested.

L125 …there was a risk…

Changed, as suggested.

L128 Explain “refuge”.

Done.

L124-131 The part about adulticiding should be specified. It is not clear whether the 150m done around confirmed case? What about 150-300?

The text is now clear that the 150m spraying was done around transit areas. We have edited this section more generally to improve readability.

L136 Why not?

A clarification has been added to the manuscript. Here, we did not include interventions related to transit areas associated with autochthonous cases outside Bergerac and the main outbreak foci, as, in this context, these areas are considered analogous to “imported” cases, with no evidence of confirmed arboviral circulation, and are therefore not relevant to the present analysis.

L118 Abbreviate the species name.

Done.

L270 Cases or numbers. Delete one.

Edited.

L408 Twice 2025. Please delete one.

Sadly, this is correct. It’s the title of the article and the year of publication combining. Hopefully, this will look better in the final journal formatting.
